# Longitudinal imaging and femtosecond laser manipulation of the liver: How to generate and trace single-cell-resolved micro-damage *in vivo*

**DOI:** 10.1371/journal.pone.0240405

**Published:** 2020-10-15

**Authors:** Daphne E. DeTemple, Sebastian Cammann, Julia Bahlmann, Manuela Buettner, Alexander Heisterkamp, Florian W. R. Vondran, Stefan K. Kalies

**Affiliations:** 1 Department for General, Visceral and Transplant Surgery, Hannover Medical School, Hannover, Germany; 2 Institute of Quantum Optics, Leibniz University Hannover, Hannover, Germany; 3 Lower Saxony Center for Biomedical Engineering, Implant Research and Development (NIFE), Hannover, Germany; 4 Deutsches Zentrum für Lungenforschung e.V. (DZL), Gießen, Germany; 5 Institute for Laboratory Animal Science, Hannover Medical School, Hannover, Germany; 6 German Centre for Infection Research (DZIF), partner site Hannover-Braunschweig, Hannover, Germany; Indian Institute of Technology Kanpur, INDIA

## Abstract

The liver is known to possess extensive regenerative capabilities, the processes and pathways of which are not fully understood. A necessary step towards a better understanding involves the analysis of regeneration on the microscopic level in the *in vivo* environment. We developed an evaluation method combining longitudinal imaging analysis *in vivo* with simultaneous manipulation on single cell level. An abdominal imaging window was implanted *in vivo* in Balb/C mice for recurrent imaging after implantation. Intravenous injection of Fluorescein Isothiocyanate (FITC)-Dextran was used for labelling of vessels and Rhodamine 6G for hepatocytes. Minimal cell injury was induced via ablation with a femtosecond laser system during simultaneous visualisation of targeted cells using multiphoton microscopy. High-resolution imaging *in vivo* on single cell level including re-localisation of ablated regions in follow-up measurements after 2–7 days was feasible. Targeted single cell manipulation using femtosecond laser pulses at peak intensities of 3–6.6 μJ led to enhancement of FITC-Dextran in the surrounding tissue. These reactions reached their maxima 5–15 minutes after ablation and were no longer detectable after 24 hours. The procedures were well tolerated by all animals. Multiphoton microscopy *in vivo*, combined with a femtosecond laser system for single cell manipulation provides a refined procedure for longitudinal evaluation of liver micro-regeneration in the same region of interest. Immediate reactions after cell ablation and tissue regeneration can be analysed.

## Introduction

The liver not only fulfils a broad spectrum of functions in the human body: from the metabolism of toxins to the synthesis of proteins and clotting factors to the storage of energy via glycogen. Several studies could also demonstrate, that the liver possesses high regenerative capacities: In the rodent model, loss of up to two thirds of the liver mass could be compensated within a week after partial hepatectomy [1–3]. This regeneration is accompanied by an augmented blood flow in the portal vein and increased DNA-synthesis in the residual hepatocytes triggering hyperplasia [2]. However, the actual pathways responsible for these reactions are not completely understood, yet. For example, blockade of regeneration of mature hepatocytes with 2-acetylaminofluorene does not stop the liver regeneration [4], suggesting precursor and/or stem cells to play a role in regeneration [5–7].

Against this background arises the need to further analyse the processes involved in liver regeneration, for example, on the cellular level to reveal the types of cells responsible for recovery of damaged regions. Ideally, the examination method would leave not only the imaged tissue unharmed, but would also allow the examined animal to stay alive for longitudinal analysis at further time points. Most standard protocols for intravital imaging of the liver involve the animal’s death after imaging [8, 9]. Cellular transport mechanisms can be visualised at very high resolution using multiphoton microscopy [10, 11]. However, the open laparotomy used in this approach leads to the animals’ death after imaging; an important limitation we sought to overcome. Therefore, we aimed for a method that not only permits the induction of targeted cell damage, but also allows the animals to stay alive after an imaging session and enables rediscovering of the damaged regions for further imaging. This would significantly reduce the number of research animals needed.

Ritsma et al. described already in 2012 [12] the feasibility of the implantation of an abdominal imaging window (AIW) for the analysis of the development of metastases. This window consisted of a titanium ring, 1.4 cm in diameter, combined with a cover glass of 170 μm thickness, which was implanted over the liver via laparotomy and secured to the skin with a purse-string suture. As described by Ritsma et al. also in subsequent publications [13–15], this window could be implanted almost anywhere in the abdominal wall and had only little influence on the animals’ wellbeing, enabling imaging for a period of up to 4 weeks [13, 14].

Multiphoton microscopy (MPM) [16] is similar to confocal fluorescence microscopy and fluorophores can also be used to mark structures of interest [17, 18]. As MPM is an application of nonlinear optical processes, intrinsic nonlinear responses of biological materials, such as autofluorescence, can also be used for imaging. Additional advantages of long incident wavelengths include low photon energies, which do not damage the material or tissue and allow a greater light penetration into the depth, and an enhanced transversal and longitudinal resolution due to the prevalence of nonlinear processes, which are restricted to the region of maximum intensity of the focused laser beam. The intensity needed for excitation is only reached in the focal point.

With this project, we are the first to combine high-resolution imaging with a method for targeted cell manipulation on a micrometre scale *in vivo* in the liver. In earlier studies, UV laser beams were used to ablate cells in abdominal tissue, for instance, in the intestine [19]. However, cell manipulation is also possible using non-linear processes. Plasma induced ablation is one of the effects caused by the interaction of ultra-short laser pulses with (organic) materials. Usually, power densities of 10^11^−10^13^ W/cm^2^ at pulse durations of 10–100 fs are needed to achieve this effect: Seed electrons are generated in the focal volume, which are accelerated and can lead to the formation of a plasma in the focal volume. This can induce precise, abrupt, and complete destruction of the tissue through the formation of a cavitation bubble [19, 20]. Due to its precision, reliability and reproducibility in homogenous materials, this process is, for instance, used for refractive surgery of the cornea [21] and is therefore very promising for targeted damage induction in other organic tissues.

**Fig 1:**
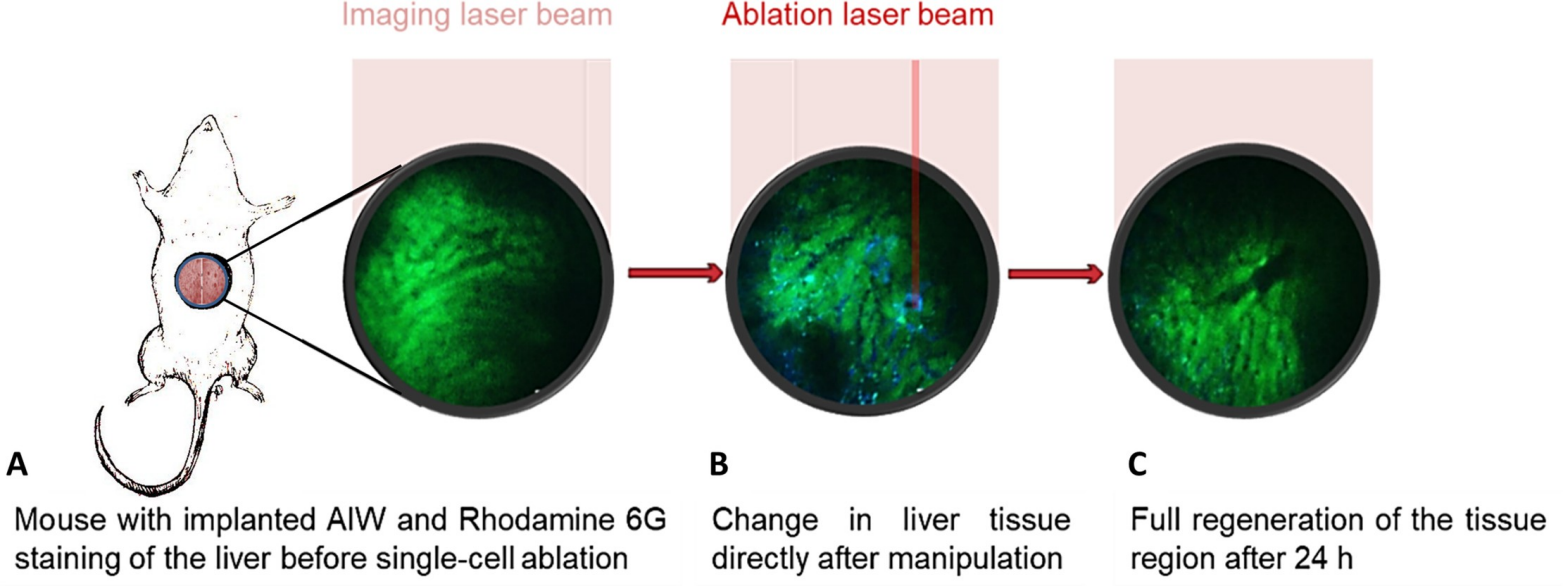
Schematic depiction of the planned procedure: (**A**) Mouse with implanted abdominal imaging window (AIW) and liver directly under the AIW. (**B**) MPM-image of the healthy liver (green: Rhodamine 6G, blue: FITC-Dextran) after application of a targeted femtosecond laser pulse. (**C**) MPM-image of a follow-up analysis of the manipulated region.

To reach our objective of establishing an optimised imaging technique for longitudinal analysis and follow-up examination of targeted tissue damage, we combined the intravital multiphoton microscopy and the implantation of an abdominal imaging window with a femtosecond amplifier ablation laser system for microscopic tissue manipulation (see Fig 1). This process allows studying of immediate and later micro-regeneration in the liver at a defined extent down to the single cell level.

## Materials and methods

### 1. Setup of the femtosecond laser systems for imaging and ablation

For the imaging setup, a Chameleon Ultra II (Coherent, US) laser system with a repetition rate of 80 MHz, tuneable for wavelengths from 680 to 1080 nm was used. The best possible imaging wavelength was determined at the beginning of measurements in an analysis with the chromophores used for staining as 780 nm. The beam was then led through an attenuator complex consisting of a λ/2-plate and a polarising beam splitter (PBS) to the first beam expansion unit (all components Thorlabs GmbH, Germany). A modified Thorlabs MPM200 microscope (Thorlabs GmbH, Germany) was used for multiphoton microscopy. Inside the microscope, the imaging beam was combined with the manipulation beam through another PBS.

The manipulation beam was produced using a Spitfire Solstice Ace laser system (Spectra-Physics, US) running at a central wavelength of 800 nm and a repetition rate of 100 Hz. The beam was led through an attenuator complex (λ/2-plate and PBS) and then through a mechanical shutter allowing for the generation of single pulses. An oscilloscope was used for control of emitted pulses. After passing through a second λ/2-plate for fine tuning of the applied laser power, the manipulation beam was coupled into the microscope’s PBS. The ablation laser power at the back aperture of objective was approximately 31–66 mW resulting in pulse energies of 3–6.6 μJ. Together, both beams passed through the microscope’s dichroic mirror, the 20x objective and into the tissue or probe. Here, the imaging beam excited the tissue’s molecules to emit light which is reflected back into the microscope and passes via the dichroic mirror to the two bandpass filters and photomultiplier tubes (PMT). The signals of the two PMTs were recorded and analysed using the imaging software ThorImageLS 1.4.

### 2. Computer-based analysis of imaging and ablation

Data was acquired using ThorImageLS 1.4 as z-stacks, with 0.02 mm layer thickness, resulting in stacks about 1–2 mm thick, or as time-stacks, with one image every 0.1 seconds, resulting in stacks of up to 50 images. Data analysis was performed using the open platform software ImageJ [22] for the determination of mean grey values and area of ablation sites. All graphs were prepared using Origin (OriginLab, US). To improve the noise ratio, we used the NOISE2VOID tool by Krull et al. [23]. This convolutional neural network was trained directly on our acquired data.

### 3. ZMTH3 cell culture

For *in vitro* testing of imaging and ablation before moving to resected organs and *in vivo* experiments, cultures of the canine pleomorphic mammary adenoma cell line ZMTH3 were used. Cells were cultured in petri dishes in supplemented RPMI medium with the selection antibiotic G418 and split when necessary (all reagents PAN-Biotech GmbH, Germany). Cells expressed mitoDsRed, a red fluorescent protein (RFP), which is tagged to mitochondria, and were stained with Calcein AM (ThermoFisher, US).

### 4. Abdominal imaging window implantation–Surgery and peri-operative management

Implantation of an abdominal imaging window was performed in 20 BALB/c mice as described by Ritsma et al. [6]. The abdominal window, consisting of a 1.4 cm titan ring and a cover glass of 1.15 cm diameter and 170 μm thickness, was prepared. Analgesia was secured by preoperative subcutaneous injection of 5 mg/kg butorphanol and directly postoperative subcutaneous injection of 5 mg/kg carprofene. Anaesthesia was induced by inhalation of 4–5% isoflurane and maintained by inhalation of 1–3% isoflurane. Eyes were covered with bepanthen or similar dexpanthenol-containing balm to prevent desiccation. During surgery, the mouse was placed on a warming mat with rectal control of body temperature. The ventral abdomen was depilated using depilatory cream and disinfected using 70% ethanol. For verification of depth of anaesthesia, the interdigital reflex was tested prior to surgery. A longitudinal laparotomy was performed starting at the xiphoid and continuing to approximately 1.5 cm caudal along the linea alba. The xiphoid was then clamped for 5 min before resection. Using a sterile cotton swab, the liver was mobilized in caudal direction and the ligamentum falciforme resected. A sterile piece of gauze was used to keep the liver in its position. The window was then attached to the liver using dots of cyanoacrylate glue and the abdomen closed around the window with a purse-string-suture. After the stop of isoflurane application, the animals were placed into new cages with fresh nesting material under a heat lamp. The animals were observed until fully awake and mobile. Soaked food and water were provided at the cage floor. The total duration of the procedure amounted to 20–30 minutes of narcosis with an average 15 minutes of surgery. After the stop of isoflurane application animals were awake and moving after about 2–5 minutes. All animals were active, climbing and feeding already hours after the operation and showed normal nesting behaviour from day 3 onwards. Each animal was kept in its own cage postoperatively and during imaging to avoid injury. To minimise pain and distress, carprofene 5 mg/kg was given until the postoperative day 3 and continued in case of symptoms. Apart from the daily determination of weight development, animals were scored for their grimace and behaviour as shown in Tables 1 and 2.

**Table 1:** Scoring of animals in experiment depending on activity.

Score	Quality	Feature	Control
1	Very active	Strong, curious, quick movements	1x per day/ 2x per week
2	Active	Curious, quick, occasional pauses of activity, up tp 10% weight loss	1x per day
3	Restricted activity	Reaction on attention, frequent pauses of activity, reddening of wounds, up to 10% weight loss	2x per day, if necessary application of carprofen
4	Calm	Indifference to surroundings, scarce activity, drowsy, reduced intake of food, purulent wounds, more than 20% weight loss	Termination of experiment
5	Lethargic	No activity, no intake of food	Not reached
6	Moribund	No activity, difficulty breathing, anticipated death	Not reached

**Table 2:** Complete postoperative scoring system for mice after implantation of AIW.

**Grimace scale: “0” = non-existent, “1” = moderate, “2” = obvious**
Opening of eyes
Swelling of nose
Swelling of cheeks
Ear position
Whiskers
**∑(Grimace)**
**Scoring: (total scoring depending on the table above)**
Weight
Nesting behaviour
Feeding behaviour
Defecation
Activity
Wound healing
Body posture
Fur
**∑ (Score)**

To achieve a comprehensive and as complete postoperative assessment of the animals as possible, the scoring system mentioned in Table 1 was extended by the mouse grimace scale as described by Langford et al. in 2010 [24], which was determined by inspection during the routine control (shown in the upper part of Table 2). The characteristics for the scoring in Table 1 were unravelled to several sub items to capture all relevant aspects, as shown in the lower part of Table 2. The worst score reached was a score of 2 (of 5, see Table 1) on day one after operation. For the postoperative scoring trend see S1 Fig.

The study was carried out in strict accordance with the recommendations in the Guide for the Care and Use of Laboratory Animals of the National Institutes of Health. The protocol was approved by the Lower Saxony State Office for Consumer Protection and Food Safety (LAVES) under the number 17/2715. All surgery was performed under isoflurane anaesthesia, and all efforts were made to minimize suffering.

### 5. Imaging using multiphoton microscopy

All animals were imaged on days 7, 8, 10 and 14 after implantation using a multiphoton microscope. An immersion objective was used with Vidisic eye gel as immersive agent. Again, induction and maintenance of anaesthesia were secured by inhalation of isoflurane. For visualisation of blood vessels, 10 mg/ml fluorescent FITC-Dextran solution combined in 100 μl of saline with 1 mM Rhodamine 6G (all reagents Sigma Aldrich, Germany) for staining of hepatocytes was injected into the warmed tail vein. During imaging, the mouse was placed on a warming mat with rectal control of body temperature and eyes were covered with bepanthen or similar dexpanthenol-containing balm. Microscopy was performed to a maximal duration of 4 hours. Every hour, an injection of 100 μl of 20% glucose-solution was given intravenously.

### 6. Single cell ablation

For the analysis of the regenerative capacities of the liver, the first imaging session was used for targeted cell manipulation in the sense of single-cell ablation. This precision is made possible by use of the above-mentioned femtosecond laser system which allows defined laser application without affecting neighbouring cells. The required laser power was determined beforehand (data shown in paragraph 4.3.2). Post-ablation imaging was continued for an hour, bevor the animals were woke up. The targeted region was marked on the titan ring to facilitate re-localisation in the follow-up imaging 1, 3 and 7 days after ablation. At the end of the last imaging session, the animal was euthanized via cardiac puncture in accordance to animal welfare regulations under continued isoflurane anaesthesia and organs resected for further analysis.

## Results and analysis

### 1. AIW implantation and concluding analysis

All in all, the animals well tolerated not only the implantation of the AIW, but also the subsequent ablation and imaging sessions. Twenty mice were included in the study, two of which died peri-operatively without detectable causes. All other animals tolerated narcosis and implantation without any complications and hardly any weight loss. Further scoring was based on the animals’ general appearance and behaviour (see Tables 1 and 2).

The total duration of the surgical procedure amounted to 20–30 minutes of narcosis and in average 15 minutes of surgery. After the stop of isoflurane application animals were awake and moving after approximately 2–5 minutes. All surviving animals were active, climbing and feeding already few hours after the operation and showed normal nesting behaviour from day 3 onwards.

Post-mortem analysis was performed on all animals at the end of the experiment two weeks after implantation. The AIW was explanted and the liver resected for further inspection via incident light- and multiphoton microscopy performed of the liver with adequate distance to manipulated regions, beneath the AIW and with contact to the gauze. No abdominal alterations were observed.

### 2. Longitudinal imaging

Of the 18 surviving animals, one developed symptoms of incomplete hemiplegia after narcosis for imaging on day 8 after implantation of the AIW. No cause was found and the animal was euthanized. All other animals showed no signs of constraints or impairments due to narcosis, ablation or handling for imaging.

For imaging on days 1, 3 and 7, the manipulated region was marked in order to render later re-localisation possible: On the titan ring and abdominal fur, rough marks for x-y-localisation were made with water insoluble pen to enable the alignment of the objective over the AIW. Upon imaging, regions with an evident and recognisable vascular structure, mostly near a larger capillary, were targeted to facilitate re-localisation in further sessions. Images from the first session were used as reference for accurate re-positioning in follow-up analysis.

Rhodamine 6G and FITC-Dextran were dissolved together in isotone saline. Narcosis was induced with isoflurane in a chamber and mice transferred to face-mask when appropriate depth of anaesthesia was reached. After pre-warming of the tail with 37°C warm water, 100 μl of the stain-mixture were injected into one of the tail veins. In nearly all cases, the duration of time needed for placing and fixing under microscope (about 5–10 minutes) sufficed for the staining to arrive in the liver. Ablated regions could be re-localised using the same targeting system as mentioned above for imaging.

**Fig 2:**
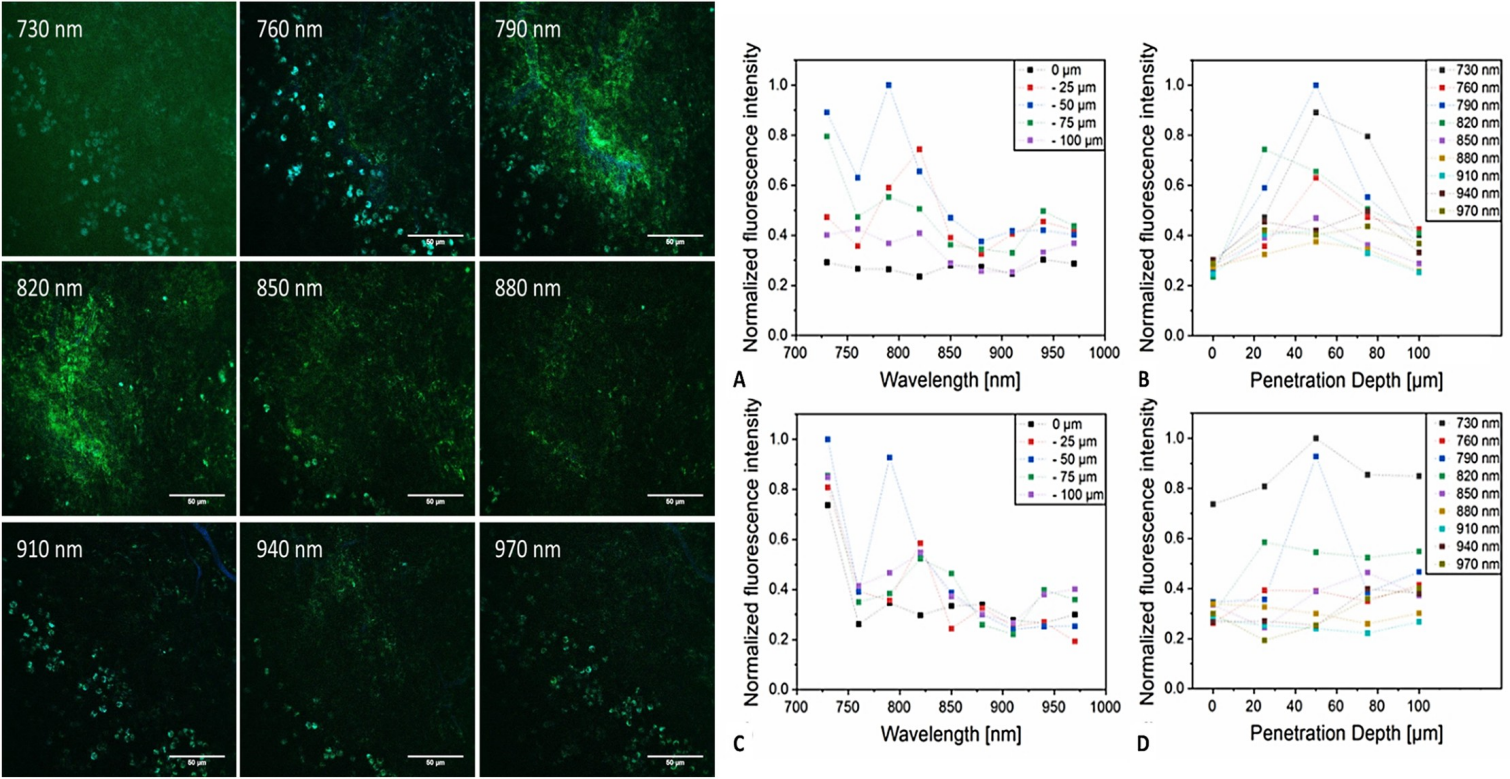
Comparison of different excitation wavelengths for imaging: [left] MPM images of the same region at the same depth in the liver at different imaging wavelengths (green = Rhodamine 6G, blue = FITC-Dextran; scale bar 50 *μ*m). [right] Diagrams considering penetration depths of different imaging wavelengths into hepatic tissue stained with FITC-Dextran (blue, graphs **A** and **B**) and Rhodamine 6G (green, graphs **C** and **D**).

In preceding measurements, the imaging parameters were analysed. For imaging, a wavelength of 780 nm was found to deliver the best possible intensities of fluorescence and contrast for our combination of staining with appropriate penetration depth into the liver tissue. For a comparison of fluorescence signals induced by different excitation wavelengths see Fig 2.

**Fig 3:**
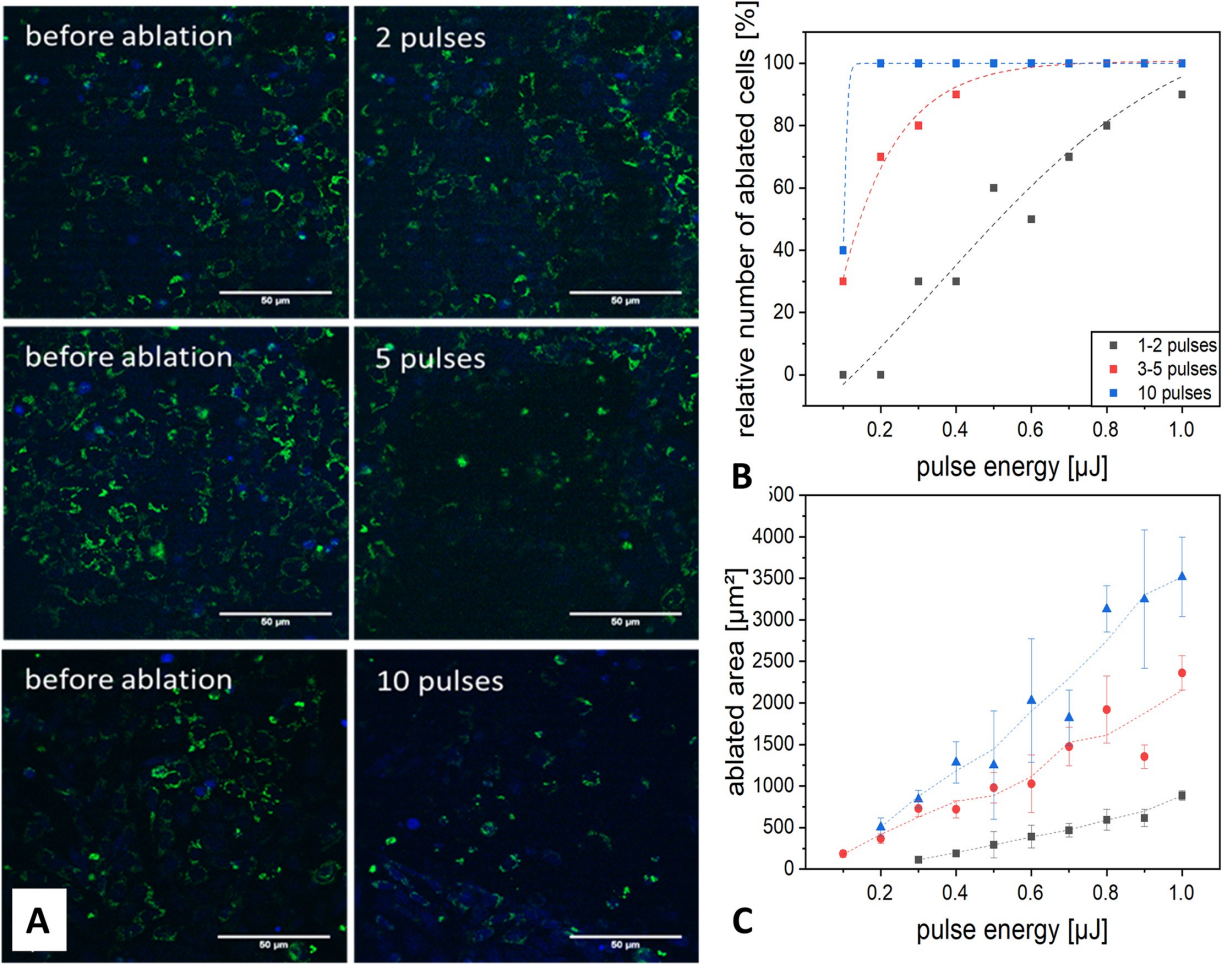
Microscopical images of the targeted ablation of ZMTH3 cells: (**A**) Expressing a mitochondrial-tagged red fluorescent protein (green) and Calcein AM (blue) using varying pulse sequences. (**B**) Dependence of the ablation efficiency on the pulse number and pulse energy based on 10 targeted cells with a sigmoidal fit function. With 10 pulses, all cells were ablated for pulse energies of more than 0.2 *μ*J. (**C**) Dependence of the ablated area on the pulse energy.

**Fig 4:**
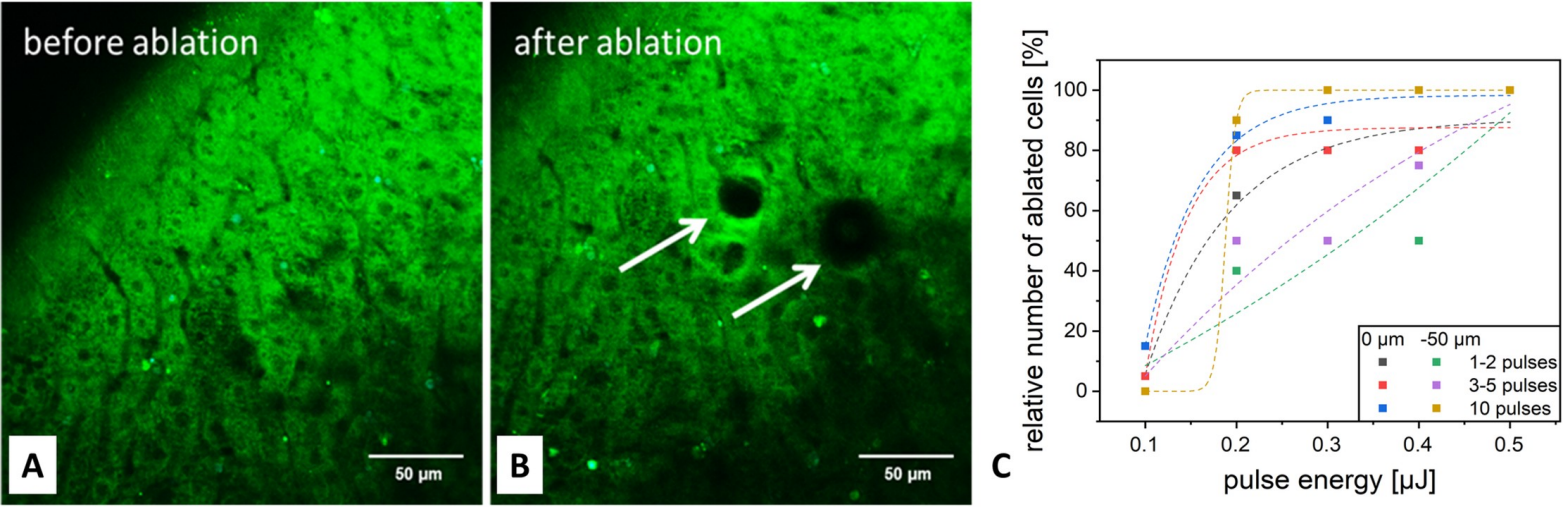
Single cell ablation *ex vivo*: (**A, B**) Visualisation of cell damage after application of the amplifier laser system. (**C**) Evaluation of the targeting efficiency.

**Fig 5:**
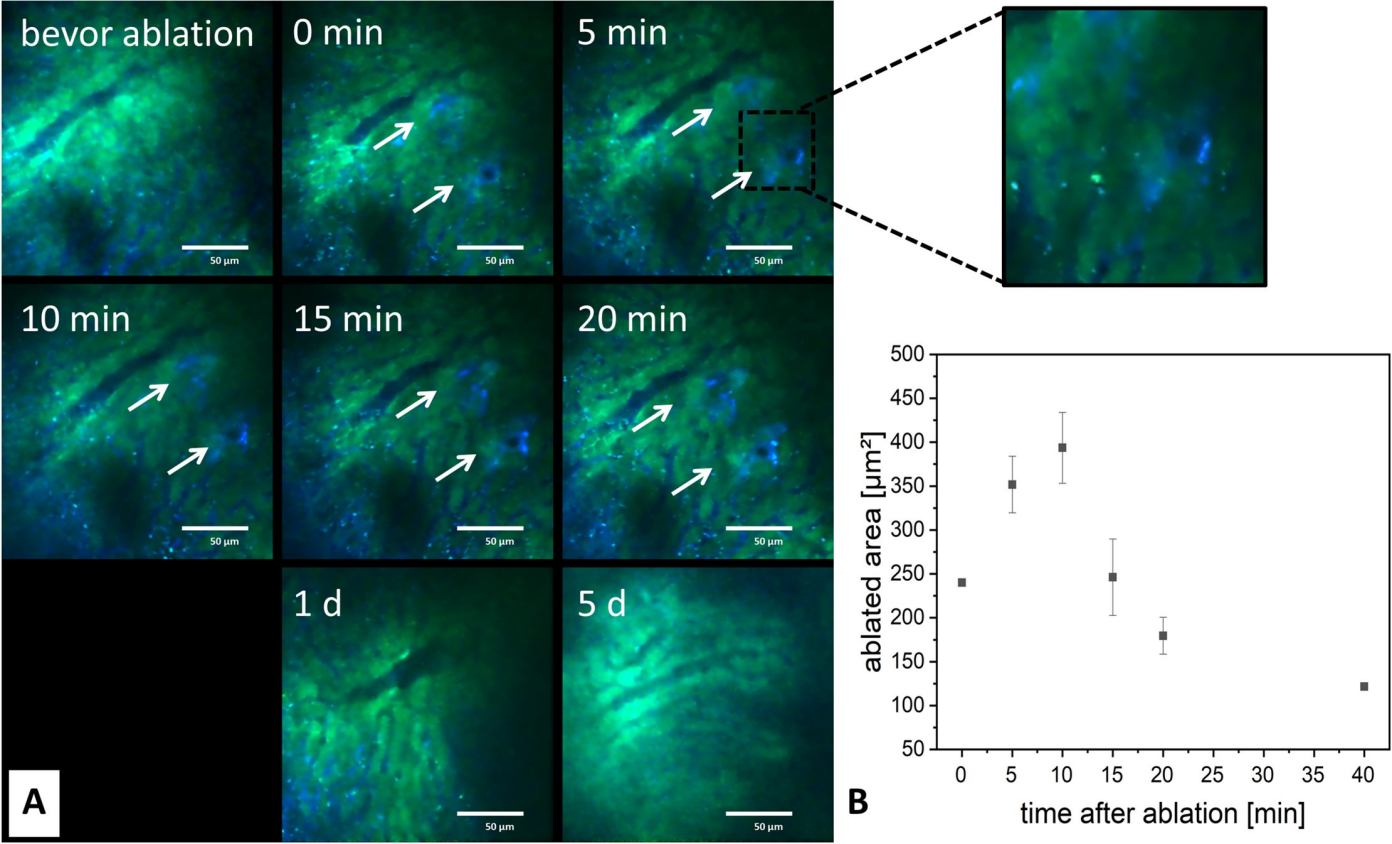
*In vivo* ablation of single cells in the liver: (**A**) Images acquired before ablation and at various time points after ablation showed fast regeneration of the targeted tissue. (**B**) The ablated area first expanded and later receded again.

For all imaging sessions, staining worked very well to sufficiently depict cells and cell structures (Rhodamine 6G) as well as tissue architecture in comparison to blood vessels/capillaries (FITC-Dextran). The success was independent of the object of investigation (cell culture, resected organ, ablation through AIW *ex* and *in vivo–*see Figs 3–5 for ablation). Via *in vivo* imaging as a time stack, the position change of intravascular structures could be observed and consecutively an estimate for the blood flow velocity calculated as 25.65 μm s^-1^ (2.565 μm in between pictures 0.1 seconds apart).

### 3. Laser ablation

In order to establish adequate ablation parameters, which would allow for precise but sufficient tissue damage, precursor experiments were performed in cell culture and *ex vivo* (see Fig 3). In cell culture, low pulse energies in the range of 0.4 μJ already led to a high percentage of ablated cells with every applied pulse train. The area, which was ablated in the field of view increased with laser power and number of pulses.

After first experiments in cell cultures, imaging and ablation were performed *ex vivo* in mice with *post mortem* implanted AIWs (see Fig 4). Intra-abdominal injection of Rhodamine 6G was used for staining of liver cells. In this setting, four pulses with a pulse energy of 0.4 μJ were sufficient at the organ surface, whereas four pulses with a pulse energy of 0.6 μJ were needed for ablation in 50 μm depth.

Ablation in the mouse *in vivo* through the AIW, after intravenous injection of Rhodamine 6G and FITC-Dextran for visualisation, required a pulse energy of 6.6 μJ to achieve sufficient ablation at 5 pulses. This value was significantly higher than those obtained in cell culture and *ex vivo*. The ablated area was smaller than in cell culture.

Directly after application of laser radiation, an enhancement of FITC-Dextran (blue channel) could be detected directly surrounding the observable microscopic tissue destruction. As FITC-Dextran is typically applied for visualisation of changes in cell permeability, one can presume that in case of manipulation of single cells with the ablation laser, this enhancement is due to damage in the cell membrane. During the further course of imaging in 5 minute intervals after ablation, the area showing increase of FITC-Dextran around the manipulated cells first expanded and then receded again (see Fig 5). The maximal expansion was reached 5 minutes after application of radiation and subsequently decreased by about 30% during the following 15 minutes. However, this increase in FITC-Dextran was not only a homogenous, areal enhancement, but also showed accumulations after 10–15 minutes similar to cellular structures. This could imply that leucocytes of the blood have migrated into the damaged tissue. The enhancement of the FITC-Dextran signal was of rather short duration: Already 40 minutes after application of radiation, the accumulation of FITC-Dextran in the targeted regions was cleared again. 24 hours later no difference in tissue architecture could be detected any more. The same applied to further long-term measurements after 1, 3 and 7 days.

## Discussion

In this study, we aimed to establish a method for targeted single-cell manipulation during longitudinal imaging for follow-up examination of the tissue micro-damage. We combined intravital multiphoton microscopy [5] through an abdominal imaging window [12] with an ablation laser system for microscopic tissue manipulation.

As already outlined above, the mechanisms involved in regeneration of the liver after tissue injury are not yet fully understood. Especially for analysis on a microscopic/cellular level, new techniques like those described in this project can help to gain further insights.

The applied dyes stained the structures intended for imaging satisfactorily, enabling the differentiation between tissue and vessels for determination of e.g. blood flow velocity. Our estimate of about 25.65 μm s^-1^ is significantly lower compared to blood flow velocities observed by other research groups calculating capillary hemodynamic in the skin with velocities of around 192 μm s^-1^ [25] or maximal velocity of portal vein flow with 69.7 mm s^-1^ [26]. This difference may be explained by the fact that our measurements concerned blood flow in liver sinusoids which should have a significantly slower blood flow compared to dermal capillaries or the portal vein. Additionally, earlier experiments by McPhee et al. [27] described cell migration to slow down blood flow in the sinusoids of anaesthetised mice and rats. Thus, the feasibility of blood flow quantification in our analyses is a promising basis for our idea to eventually evaluate the change in blood flow after ablation and during the course of regeneration.

With multiphoton microscopic imaging through an AIW, longitudinal imaging of all organs and/or tissues accessible from the abdominal (or thoracic) wall, such as liver, bowel, stomach, lungs, etc., is possible. Furthermore, dynamic imaging is possible in the acute and long-term setting: Alterations made to cellular integrity or tissue structures can be observed and recorded simultaneously. By implantation of the abdominal window, a closed system was achieved, which insured the animals’ survival and made longitudinal analysis after ablation possible. We could verify the observations described by Ritsma et al. [13] for AIW implantation: The mice tolerated the implantation of the titanium and glass windows without any significant restraints. This allowed for the next step, the longitudinal evaluation of the processes following micromanipulation via imaging of the same region over the course of several days as re-localisation via external targeting marks and vascular microstructure was possible. Even though MPM imaging was performed over a period of up to one hour at a time, we did not experience any significant photobleaching of the dyes used.

For ablation in ZMTH3 cell culture as well as livers ex vivo (through an AIW), an average of 5 pulses at 0.5 μJ pulse energy sufficed for manipulation of cells, whereas 5 pulses at 6 μJ pulse energy were needed for sufficient ablation in livers in vivo, corresponding to energy 12 times as high. This difference might be caused by the necessary penetration depth and by the surrounding tissue. It is however unlikely, that massive energy transport during the pulse by blood flow or cell transport occurs, as our high pulse energy femtosecond laser pulses induce nonlinear interactions such as multiphoton ionization but do not lead to an accumulation of heat.

After ablation *in vivo*, the maximal areal extent of FITC-Dextran enhancement was reached after 5 minutes, reduced by 30% after 20 minutes and was no longer detectable after 40 minutes. This very dynamic process clearly indicated that the tissue was ablated and not only photobleached. For cellular, FITC-positive reactions, the maximum was reached after 15 minutes, indicating a slightly slower reaction time of cellular invasion as compared to instantaneously increased permeability of injured cells. These reactions as well were no longer detectable 40 minutes after ablation. In controls 1, 3 and 7 days after manipulation, no change in tissue structure was observed. These findings imply rapid micro-regenerative reactions, clearing up for example cell debris, leading to the restitution of the tissue architecture when the extent of injury was only on a micrometre scale. These apparently very quick reactions are highly interesting and should be analysed in more detail by continuous imaging in the first minutes after ablation.

Studies have shown liver regeneration upon acute injury of small parts of the liver mass to induce regeneration in form of hypertrophy, mediated mainly by mature, periportal hepatocytes [4, 28]. We therefore suspect the targeted ablation of single cells to induce phenotypic changes in the surrounding local hepatocytes, which react to the altered microenvironment via regeneration mechanisms, like cell hypertrophy, proliferation and/or cellular rearrangement. Apoptosis of the ablated cells induce inflammation, which mediates the recruitment of intrahepatic immune cells. Hepatic stem cells would be expected to be activated upon enduring or chronic liver injury, which cannot be compensated by local reactions of mature hepatocytes [28, 29]. As our damage is localised micro-damage and hepatocytes are not hindered in proliferation, we assume them to be the major player in the regeneration. Apart from the mentioned time aspects, the fact that no alterations could be detected in the follow-up indicates that the extent of ablation was not sufficiently large to induce lasting injury to the organ tissue. In future experiments, ablation could therefore be extended to larger regions targeting more than only single cells. This might on the one hand make portal ablation versus central ablation hard to distinguish, as no clear borders exist between these regions and tissue damage will probably spread after initial manipulation. On the other hand, more damaged cells will induce more regenerative reactions making these easier to detect, qualify and even quantify.

The targeting of more detailed ablation in the lobule would potentially allow the analysis of the direction of cell migration in the course of regeneration and a possible involvement of stem- and progenitor cells. Furthermore, another interesting aspect would be the comparison of regenerative mechanisms upon different kinds of tissue injury: Targeted laser-based micro-manipulation versus “conventional” mechanisms such as toxins (CCl_4_), mechanic damage (partial hepatectomy) or hypoxia (warm ischemia). Analysis of reactions on these mechanisms would also enable an evaluation of the influence of local (cell level) versus systemic (organ level) injury.

## Supporting information

S1 Fig Overview of animal scoring over the duration of the experiments: (**A**) Average weight development after implantation of an AIW during the course of experiments. (**B**) Development of different scores (see Tables 1 and 2 above) after implantation of an AIW during the course of experiments (average over all animals, error bars = standard deviation).
(TIF)

S1 File (XLSX)
