# Supplementary figures and images for: Longitudinal imaging and femtosecond laser manipulation of the liver: How to generate and trace single-cell-resolved micro-damage in vivo
Source: PLoS One. 2020 Oct 15;15(10):e0240405. doi: 10.1371/journal.pone.0240405 (PMC7561146; doi:10.1371/journal.pone.0240405)

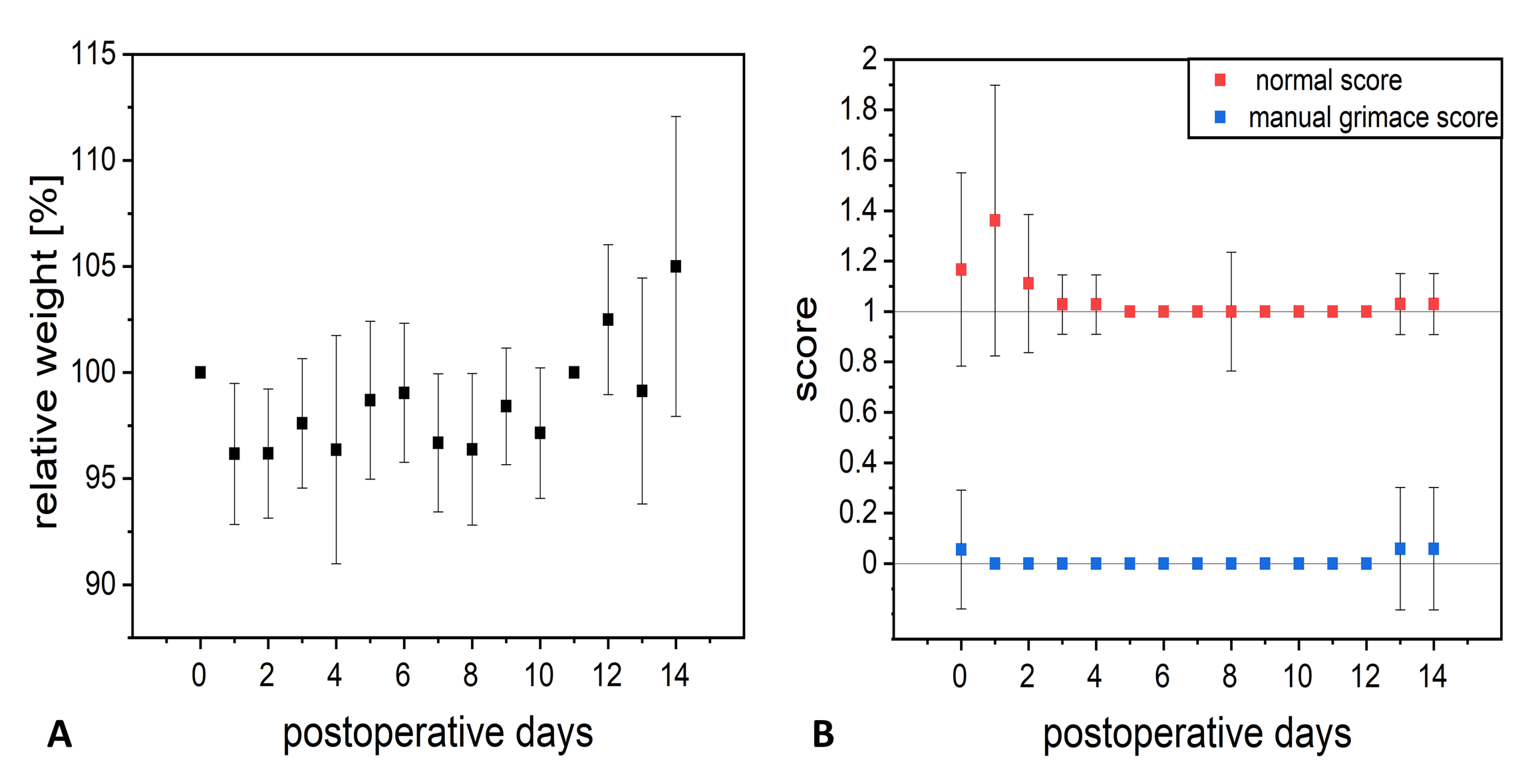

Supplement: S1 Fig — Overview of animal scoring over the duration of the experiments: (A) Average weight development after implantation of an AIW during the course of experiments. (B) Development of different scores (see Tables 1 and 2 above) after implantation of an AIW during the course of experiments (average over all animals, error bars = standard deviation). (TIF) [file pone.0240405.s001.tif]
